# Radiation-induced upregulation of telomerase activity escapes PI3-kinase inhibition in two malignant glioma cell lines

**DOI:** 10.3892/ijo.2013.1970

**Published:** 2013-05-31

**Authors:** P. MILLET, C. GRANOTIER, O. ETIENNE, F.D. BOUSSIN

**Affiliations:** 1 CEA, DSV-IRCM-SCSR, Laboratory of Radiopathology, UMR 967, F-92260 Fontenay-aux-Roses;; 2 INSERM, UMR 967, F-92260 Fontenay-aux-Roses;; 3 Univ Paris Diderot, Sorbonne Paris Cité, UMR 967, F-92260 Fontenay-aux-Roses;; 4 Univ Paris-Sud, UMR 967, F-92260 Fontenay-aux-Roses, France

**Keywords:** telomerase, radiation, PI3-kinase, radiosensitization, glioma, glioblastoma

## Abstract

Tumor relapse after radiotherapy is a great concern in the treatment of high-grade gliomas. Inhibition of the PI3-kinase/AKT pathway is known to radiosensitize cancer cells and to delay their DNA repair after irradiation. In this study, we show that the radiosensitization of CB193 and T98G, two high-grade glioma cell lines, by the PI3K inhibitor LY294002, correlates with the induction of G1 and G2/M arrest, but is inconsistently linked to a delayed DNA double-strand break (DSBs) repair. The PI3K/AKT pathway has been shown to activate radioprotective factors such as telomerase, whose inhibition may contribute to the radiosensitization of cancer cells. However, we show that radiation upregulates telomerase activity in LY-294002-treated glioma cells as well as untreated controls, demonstrating a PI3K/AKT-independent pathway of telomerase activation. Our study suggests that radiosensitizing strategies based on PI3-kinase inhibition in high-grade gliomas may be optimized by additional treatments targeting either telomerase activity or telomere maintenance.

## Introduction

Glioblastoma multiforme (GBM) is the most common and the most aggressive brain tumor with a median survival of only 15 months (1,2). Despite conjugated surgery, radiotherapy and chemotherapy most patients die within the first year of diagnosis (3,4). The molecular mechanisms implicated in the resistance of glioblastoma to chemotherapies and radiotherapies overlap with those implicated in oncogenesis (5). Among those, the PI3K/AKT pathway which is implicated in regulation of cell proliferation, cell cycle, survival, apoptosis, migration and angiogenesis, is a major one (6–16).

The activation of the AKT pathway promotes the transition from anaplastic astrocytoma to glioblastoma (17), is correlated to histological malignant evolution and is a negative prognosis factor (18,19). Moreover, the intrinsic radioresistance of glioblastoma is correlated with activation levels of AKT (15) and the activation of AKT confers them radioresistance (7). During carcinogenesis, the activation of the AKT pathway mainly occurs by the gain of activity of upstream activators such as EGFR (12,20–23), or by the loss of activity of an upstream inhibitor, PTEN (7,24,25). PTEN dephosphorylates PIP3 into PIP2 via its lipid-phosphatase activity and decreases the level of the phosphorylated active form of AKT (24,26).

During gliomagenesis, the AKT pathway is also frequently activated (27,28) and PTEN disrupted (29–31). Consequently the inhibition of AKT by either PTEN re-expression or PI3K inhibitors impairs DNA repair and radiosensitizes glioblastoma (13,15,32,33).

Telomerase is a specific reverse transcriptase that elongates the telomeres, enables unlimited proliferation of cancer cells and is currently related to their radioresistance (34–36). Consequently telomerase inhibition shortens telomeres and radiosensitizes cells (37). Telomerase is reactivated in 80–100% of glioblastomas (38) and its levels are correlated with the pathological grade and the prognosis of the tumor (38–42). This suggests that telomerase might also intervene in the radioresistance of glioblastomas by either triggering telomere maintenance and/or chromosome healing (43). Consequently telomere targeting or telomerase inhibition radiosensitizes glioblastoma cell lines (11,44–46). The evidenced importance of telomerase activity in the biology and the clinical outcomes of gliomas points out this enzyme as an appropriate therapeutic target for the radiosensitization of glioblastomas.

Interestingly, the telomerase activity is directly regulated by AKT either by phosphorylation of the hTERT subunit (47) or by both post-translational and transcriptional mechanisms (48,49). Furthermore, ionizing radiation increases the telomerase activity in various cancer cell lines (35,50–53) by a post-translational mechanism implicating PI3K/AKT pathway (54). But still, the upregulation of telomerase activity induced by ionizing radiation in glioblastoma cells (46) remains to be linked to PTEN/PI3-kinase/AKT pathway.

As both PI3K/AKT and telomerase appear to be potential targets for cancer therapy and radio-sensitization of brain cancers (5,11,15,16,43,45,55–57), we decided to study the links between telomerase activity and AKT pathway in human glioblastomas in order to challenge the idea of a ‘killing two birds with one stone’ radio-sensitizing strategy.

Therefore, we evaluated the effects of a specific PI3K inhibitor (Ly-294002) (58) in the radioresponse of two telomerase positive high-grade glioma cell lines: CB193 (grade III WHO) a PTEN null one (59,60) and a T98G (grade IV WHO) a PTEN harbouring one (61,62).

## Materials and methods

### Cell culture

Human malignant glioma cell lines CB193 (astrocytoma, grade III) (59) and T98G (glioblastoma multiforme, grade IV) (61,62) were kindly provided by Dr G. Gras (CEA, France). Cultures (5×10^5^ cells/flask) were maintained in DMEM medium (Life Technologies, Grand Island, NY, USA) supplemented with 10% fetal bovine serum (Life Technologies), 2 mM glutamine (Sigma, St. Louis, MO, USA) and antibiotics (penicillin, 100 U/ml and streptomycin, 100 *μ*g/ml; Sigma), in a 5% CO_2_ atmosphere at 37°C. Cells were collected by trypsin treatment and counted using trypan blue. Ly-294002 (Ly, Biomol) a potent inhibitor of phosphoinositol 3-kinase (PI3K) was dissolved in DMSO (Sigma) and stored at −20°C. This solution was diluted in culture medium 24 h after seeding to treat cultures during exponential asynchronous growth to a final concentration of 50 *μ*M. Control cells were treated with the corresponding concentration of DMSO (0.2%). Cells were γ-irradiated during exponential asynchronous growth at 2 or 5 Gy (IBL637, CisBio International). When cells were treated with PI3K inhibitor and γ-irradiated, 50 *μ*M of Ly-294002 was added to culture medium 1 h before irradiation.

### Western blot analysis

Cells were lysed in ice-cold CHAPS lysis buffer. The protein concentration was estimated in the supernatant using the Bio-Rad protein assay according to the manufacturer’s protocol. Lysates (30 *μ*g for CB193 and 25 *μ*g for T98G) were separated by SDS-PAGE under reducing conditions before transfer onto nitrocellulose membranes (Life Technologies). Equal protein loading was confirmed by Ponceau staining. Blots were blocked in TBS buffer containing 5% non-fat dried milk for 1 h at room temperature. The membranes were incubated for 1 h at room temperature or overnight at 4°C with the primary antibodies: rabbit anti-γ-AKT Ser473 clone 193H12 (Cell Signaling Technology, Danvers, MA, USA), mouse anti-AKT (Cell Signaling), mouse anti-PTEN clone A2B1 (Becton-Dickinson, Franklin Lakes, NJ, USA) or mouse anti-β-actin (Sigma). Membranes were then washed and incubated with the secondary antibody (GE Healthcare, Velizy, France) for 1 h at room temperature before washes. Detection of antibody binding was performed by enhanced chemiluminescence according to the manufacturer’s instructions (ECL Super Signal Western blotting detection kit, GE Healthcare).

### Colony-forming unit (CFU) assay

For CFU assay, CB193 and T98G (5×10^5^ cells/T25 flask) were cultured for 24 h at 37°C then treated with Ly-294002 or the corresponding concentration of DMSO (Sigma) and γ-irradiated as described above. Cultures were incubated at 37°C for another 24 h. Cultures were then trypsinized and counted using Trypan blue. A fixed number of experimentally determined living cells (600 cells for T98G, 800 cells for CB193) were re-seeded in 6-well plates in fresh culture medium without PI3K-inhibitor and CFU (>50 cells) were stained with methylene blue and counted after 14–20 days in culture.

### Apoptosis assay

Apoptotic cells were quantified by the detection of cleaved capsase-3 by immunostaining. Briefly, cells were grown in 8-well Lab-Tek chamber slides and fixed in 4% paraformaldehyde and permeabilized using 0.1% Triton X-100 and 0.1% sodium citrate. After a blocking step (7.5% goat serum and 7.5% fetal calf serum in PBS, 1 h at room temperature), cells were incubated with a 1:200 dilution of rabbit antibody specific for the cleaved form of caspase-3 (cleaved caspase-3 (Asp175) antibody, Cell Signaling) for 1 h at room temperature. After washings, cells were incubated with 1:125 dilution of Texas-Red-conjugated anti-rabbit IgG for 50 min at room temperature and then counterstained with DAPI before observation under a fluorescence microscope (Olympus BX51).

### Cell cycle analysis

Cells were collected by trypsin, washed with PBS, fixed in 80% ethanol and kept at −20°C for ≥24 h. They were then washed in PBS and resuspended in 50 *μ*g/ml propidium iodide and RNase-DNase free (10 *μ*g/ml). The cell suspension was incubated for 30 min at room temperature and cell cycle distribution was determined by flow cytometry (FACSCalibur, BD, Franklin Lakes, NJ, USA), with CellQuest software analysis and quantification using Win-MDI software.

### Immunostaining

Cells were grown in 8-well Lab-Tek chamber slides and fixed in 4% paraformaldehyde and permeabilized using 0.1% Triton X-100 and 0.1% sodium citrate. After a blocking step (7.5% goat serum and 7.5% fetal calf serum in PBS, 1 h at room temperature), cells were incubated with the primary antibody: mouse anti-γ-H2AX clone JBW301 (Merck Millipore, MA, USA), diluted in blocking buffer (1:200) for 1 h at room temperature. Then, cells were washed and incubated with Alexa-594 anti-mouse antibody (Life Technologies) diluted in blocking buffer (1:400) for 50 min at room temperature. After washing, cells were then counterstained with DAPI before observation under a fluorescence microscope (Olympus BX51).

### Telomerase activity assay

Telomerase activity was assessed with the TRAPeze ELISA Telomerase Detection kit (S7750, Merck Millipore) according to the manufacturer’s instructions. Briefly, the cells were seeded (2×10^6^ cells/T75 flask) for 24 h at 37°C then treated with Ly-294002 or the corresponding concentration of DMSO and γ-irradiated as described above. Cultures were transferred to an incubator at 37°C for another 24 h. Then the cells were collected by trypsin treatment in cold PBS and counted in triplicate using trypan blue. Cells were lysed in ice-cold CHAPS lysis buffer. After incubation at 4°C for 30 min and a centrifugation at 16,000 g for 25 min at 4°C, cell extracts were kept frozen at −80°C. Telomerase activity was then measured on proteins corresponding to an experimentally fixed number of cells (234 cells CB193 and 166 cells for T98G) in a 50-*μ*l reaction mixture containing 10 *μ*l of 5X TRAP reaction mix and 2 U of *Taq* DNA polymerase (GE Healthcare). The reaction mixture was incubated for 30 min at 30°C. The extended products were amplified by a polymerase chain reaction (PCR, 32 cycles at 94°C for 30 sec and at 59°C for 30 sec) on a PTC-200 thermocycler (MJ Research). The amplification products were immobilized onto streptavidin-coated microtitre plates and detected by an anti-DNP antibody conjugated to horseradish peroxidase (HRP). After addition of the peroxidase substrate (3,3′, 5, 5′-tetramethylbenzidine), the amount of TRAP products was determined by measuring the absorbance at 450 and 690 nm. Telomerase activity was semi-quantified using an internal standard curve.

### Statistical analysis

All statistical analyses were performed using the StatView software (Abcus Concepts) and Student’s t-test was used to evaluate the statistical significance of mean values between conditions. In each figure error bars represent standard error of the mean and statistical significance levels are noted as follows: ^*^P<0.05, ^**^P<0.01, ^***^P<0.001.

## Results

### Ly-294002 radiosensitizes glioma cell lines

**Figure 1:**
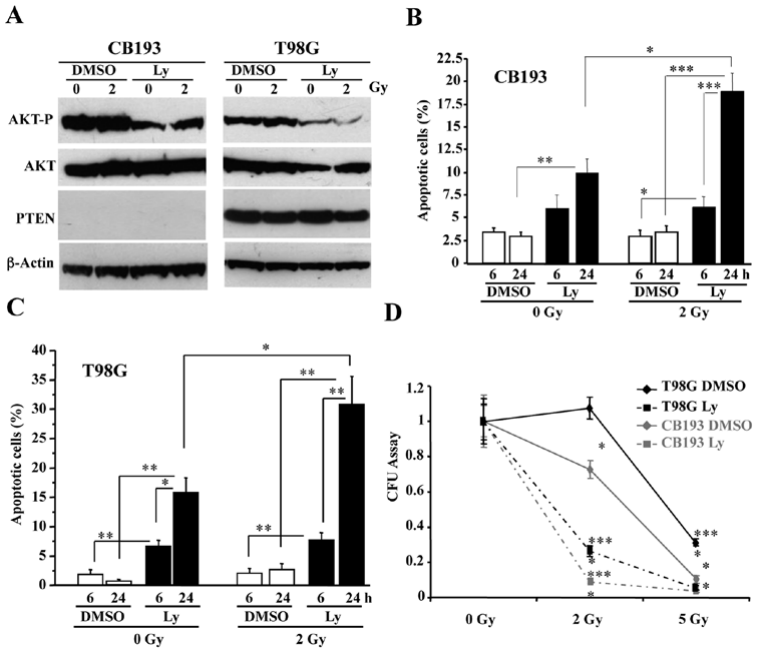
Ly-294002 radiosensitizes CB193 and T98G. (A) Western blot analysis of AKT, AKT-P (phosphorylated form of AKT), PTEN and β-actin, 24 h after irradiation when CB193 and T98G were pre-treated with Ly-294002 or DMSO. (B and C) Cleaved caspase-3 detection by immunofluorescence 6 and 24 h after irradiation. Histograms showing the percentage of cleaved caspase-3-positive cells ± standard deviation with the respect to the total DAPI stained CB193 (B) and T98G (C) populations. Results are representative of two independent experiments (>400 cells analyzed per condition). (D) Colony forming unit (CFU) assay on CB193 and T98G treated with PI3K inhibitor (50 *μ*M Ly294002) and irradiated with 2 or 5 Gy. A fixed number of living cells were seeded in plates with fresh culture medium without PI3K inhibitor 24 h after irradiation and colonies (>50 cells) were counted 14–20 days later. Mean number of colony forming unit from triplicate cultures ± standard deviation, are representative of two independent experiments. The curves were normalized to that of sham-irradiated control DMSO-treated cells. Statistics (t-test): ^*^P<0.05; ^**^P<0.01; ^***^P<0.001.

As shown in Fig. 1A, treatment with 50 *μ*M Ly-294002 resulted in a significant dephosphorylation of AKT in both CB193 and T98G glioma cell lines, but 2-Gy radiation had no detectable effect on AKT phosphorylation. Consistent with the importance of AKT phosphorylation for cell survival, immuno-detection of cleaved-caspase-3 showed that apoptosis increased in Ly-294002-treated cultures (Fig. 1B and C). Moreover, 2-Gy radiation did not significantly induce apoptosis in DMSO-treated glioma cell lines, but nearly doubled apoptosis levels in Ly-294002-treated cells 24 h after irradiation (PI) (30.9±4.6 vs 15.7±2.6% in T98G cells and 18.9±2.0 vs. 9.2±1.5% in CB193 cells), showing that Ly-294002 radiosensitizes glioma cell lines.

This was further confirmed by determining the capacity of irradiated glioma cells to form colonies after a 24 h treatment with 50 *μ*M Ly-294002 or with DMSO in a CFU assay (Fig. 1D). Ly-294002 strongly decreased the clonogenicity of 2-Gy-irradiated CB193 and T98G cells, whereas 2-Gy radiation alone had no (T98G) or only a moderate (CB193) effect on DMSO-treated glioma cell clonogenicity. Radiosensitization by Ly-294002 was also observed in T98G cells after 5 Gy, a dose that was sufficient to abolish CB193 clonogenicity.

### Radiation-induced G2/M arrest in Ly-294002-treated glioma cells

The PI3K/AKT pathway plays multiple roles in cell cycle progression (63). Measuring DNA content by flow cytometry showed that Ly-294002 induced a G1 arrest in glioma cells, consistently with the requirement of PI3K/AKT pathway for G1/S transition that has been previously reported in many cell types (63).

**Figure 2:**
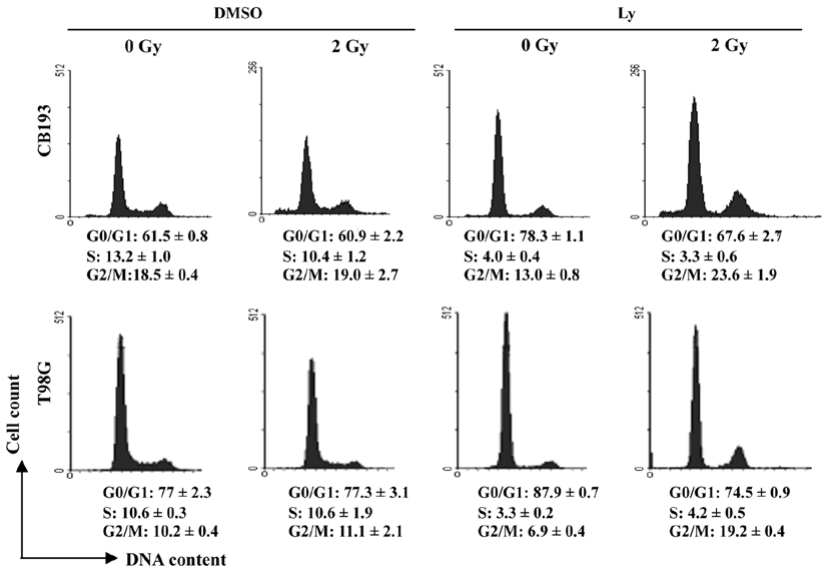
Ly-294002 induces a G2/M cell cycle arrest in irradiated T98G and CB193. Histograms of the 24-h cell cycle of surviving CB193 and T98G treated with 50 *μ*M Ly and irradiated at 2 Gy and controls. The cells were stained with propidium-iodide and analysed by FACS. The percentages of cells in different phases of the cell cycle from triplicate cultures are expressed with respect to the total number of viable cells (corresponding to an analysis of 10^5^ cells) and are representative of two independent experiments performed 24 h after irradiation.

Consistent with the little or absent effect of 2-Gy radiation on glioma cell viability, as shown above (Fig. 1D), the cell cycle progression was not altered in irradiated DMSO-treated cells (Fig. 2). Besides, a significant decrease in S phase cells showed that Ly-294002 blocked G1/S transition in irradiated cultures similarly to the non-irradiated ones. Moreover, irradiation induced an increase in G2/M cells in Ly-294002-treated cultures, which was more pronounced in T98G than in CB193 cells. These data revealed that, besides its effects at the G1/S transition, Ly-294002 also inhibited cell cycle progression at the G2/M transition after radiation-induced DNA damage.

### Ly-294002 delays DNA double strand break (DSB) repair

**Figure 3:**
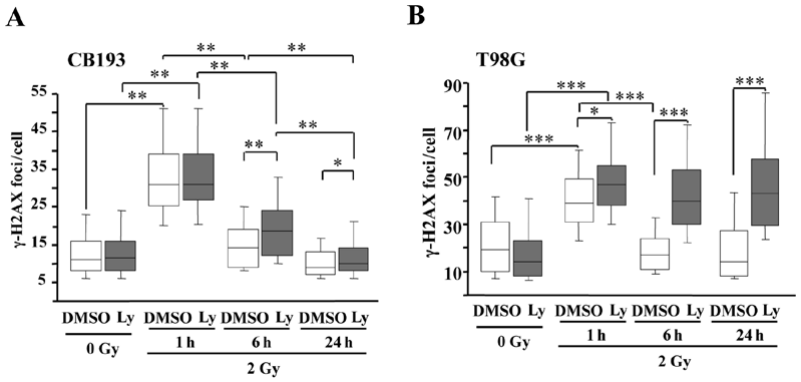
Ly-294002 delays diversely the DNA repair in T98G and CB193. Box graphs showing the distribution of γ-H2AX foci per cell in CB193 (A) and in T98G (B) cells 1, 6 and 24 h after irradiation (200–400 nuclei analyzed per condition). Boxes include 50% of the values centered on the median (the horizontal line through the box). The vertical lines begin at the 10th percentile and end at the 90th percentile. Results are representative of two independent experiments. More than 200 nuclei per condition in at least three different fields were counted. Statistics (t-test): ^*^P<0.05; ^**^P<0.01; ^***^P<0.001.

DNA damage and repair can be evaluated by quantifying γ-H2AX nuclear foci (64,65). H2AX is a member of the nucleosome core histone H2A family, which is recruited and phosphorylated on serine 139 in chromatin surrounding the site of double strand breaks (DSBs) by kinases of the PI-3K family, ATM, DNA-PKcs or ATR (66,67). In both CB193 and T98G cells, 2-Gy irradiation induced a significant increase in γ-H2AX foci at 1 h PI, which returned to basal levels at 6 h PI, revealing no difference in the kinetics of DNA repair between the two glioma cell lines. Ly-294002 did not modify the number of γ-H2AX foci at 1 h PI in irradiated cells (Fig. 3). This confirms that PI3K inhibition does not prevent DSB signaling at the concentration we used in agreement with previous studies (13,68). By contrast, Ly-294002 inhibited the decrease in γ-H2AX foci in irradiated T98G cells at 6 and 24 h PI, suggesting that PI3K inhibition suppressed DSB repair. Ly-294002 had smaller effects on CB193 since the number of foci was only slightly increased at 6 h PI in Ly-294002-treated cells compared with DMSO treated controls and recovered its basal level at 24 h PI. Altogether these data evidenced difference in the effects of Ly-294002 on DNA repair between the two cell lines. As we have shown above, the compound had similar effects on apoptosis induction and clonogenicity of the two glioma stem cells after irradiation, thus our data suggest that the radiosensitization by Ly-294002 is not strictly related to its effects on DNA repair.

### Ly-294002 does not prevent radiation-induced upregulation of telomerase activity

**Figure 4:**
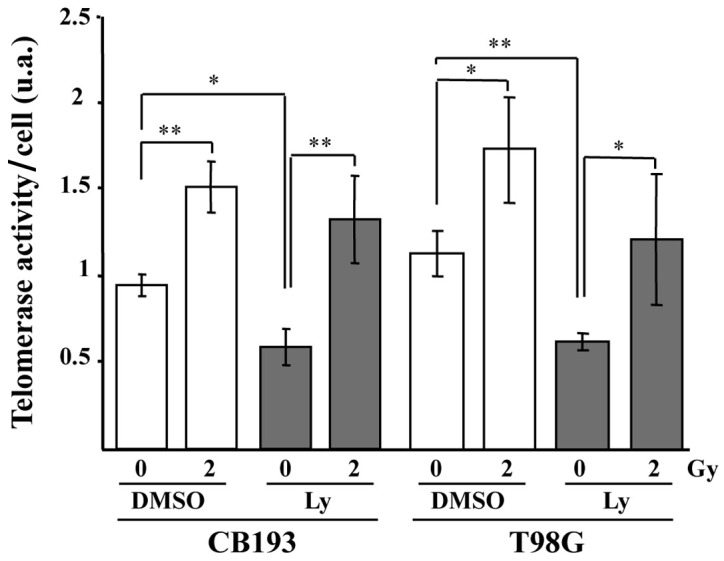
Influence of Ly-294002 treatment on telomerase activity. TRAP assay was performed on proteins corresponding to a fixed number of cells 24 h after irradiation. Cell related telomerase activity from duplicate ± standard deviation is representative of two and four independent experiments for CB193 and T98G, respectively. Statistics (t-test): ^*^P<0.05; ^**^P<0.01; ^***^P<0.001.

PI3K inhibition induced by Ly-294002 decreases the telomerase activity (Fig. 4) and dephosphorylates AKT in both sham-irradiated CB193 and T98G, suggesting that telomerase activity could be regulated by PI3K and AKT phosphorylation in glioblastomas, as in many cell types (47,49). Therefore, PI3K/AKT seems to regulate at least partly basal telomerase activity in our model.

We also found that radiation significantly increased telomerase activity in both CB193 and T98G at 24 h PI (Fig. 4). However, whereas Ly-294002 significantly decreased telomerase activity in unirradiated glioma cells, it failed to prevent the radiation-induced increase in telomerase activity in irradiated cells, ruling out a role of the PI3K/AKT pathway in the radiation-induced upregulation of telomerase activity in our model.

## Discussion

The PI3-kinase/AKT pathway is more and more regarded as an interesting therapeutic target for the radiosensitization of glioblastoma, but the mechanisms of radiosensitization resulting from the inhibition of the PI3K/AKT pathway remain still unclear. Its inhibition has been reported to impair DNA repair in glioblastoma cells following ionizing radiation, thereby blocking cell cycle progression and cell death (13). In this study, we have shown that the radiosensitization of two glioma cell lines by the PI3K inhibitor, Ly-294002, correlated with the induction of G1 and G2/M arrests, but was inconsistently linked to a delayed DSBs repair. The PI3K/AKT pathway has been also shown to activate radioprotective factors such as telomerase, which inhibition may contribute to radiosensitization (11,44–46). However, we have shown that radiation upregulated telomerase activity in Ly-294002-treated glioma cells as well as in untreated controls, regardless of their PTEN status, evidencing a PI3K/AKT independent pathway of telomerase activation. High-grade gliomas are known for their inter- and intra-patient heterogeneity. They express diversely telomerase activity and telomerase sub-units, but this expression is strongly correlated to their progression in malignancy and a poor clinical outcome (38,39,42,69–71). Our study tends to indicate that the strategy of radiosensitization of high-grade gliomas should combine different approaches and should be adapted to the individual characteristics of the tumor especially regarding their telomerase status.

Numerous previous reports have shown that inhibition of the PI3K/AKT pathways radiosensitize gliomas (13,15,32,33), consistently with the activation of PI3K/AKT conferring radioresistance (7). Ionizing radiation has been shown to increase Akt phosphorylation in various cell lines including gliomas (32,72). However, we did not find any radiation-increase of AKT phosphorylation in our two glioma cells, consistently with the study by Li *et al*(32) showing that AKT phosphorylation occurred only in a subset of glioblastoma cells.

Ly-294002 induced a G1 arrest in both CB193 and T98G cells in accordance with the importance of the PI3K/AKT signaling for G1/S transition (73–75). Moreover, as previously reported in other cell lines (76,77), inhibition of the PI3K/AKT pathway resulted in an accumulation in G2/M phase, but only after irradiation. Inhibition of the PI3K pathway has been shown to impair DNA repair after ionizing radiation, suggesting that the blocking at the G2/M transition and subsequent cell death may result from an inhibition of DSB repair (13,78). However, this is not fully sustained by our present study showing that the G2/M arrest was correlated with a delay in DSBs repair only in T98G but not in CB193 cells, after the treatment with Ly-294002. Activation of AKT has been also shown to promote G2/M transition through the activation of downstream molecules such as cyclin B associated kinase, NF-Y, Chk1 and FOXO3A (79–81). Our data suggest that beside possible inhibition of DNA repair depending on the cellular context, Ly-294002 inhibits the signaling pathway required to pass the G2/M checkpoint independently of DNA repair completion in irradiated cells.

Irradiation has been shown to upregulate telomerase activity in various cell lines (35,50–53) including a glioblastoma cell line (46). AKT is able to phosphorylate hTERT, the catalytic subunit of telomerase and activate telomerase activity (47). Recently, AKT has been also shown to facilitate nuclear import of hTERT (82). Moreover, ionizing radiation has been reported to upregulate telomerase activity in cancer cell lines by post-translational mechanism via the PI3K/AKT pathway (54). While Ly-294002 decreased telomerase activity in unirradiated CB193 and T98G cells, concomitantly with AKT dephosphorylation and G1 arrest, we have shown that it did not prevent the radiation-induced increase of telomerase activity, which was not correlated with an increase of AKT phosphorylation in these cell lines. These results rule out a predominant role of the PI3K/AKT pathway in the radiation-induced upregulation of telomerase activity in our glioma cells lines suggesting that an alternative pathway is involved which remains to be determined. Such AKT/PKB independent upregulation of telomerase activity after irradiation have been already observed in other cell lines (83) but related to delayed DSB repair. Complementary studies of DSB repair-related molecules are needed in our model.

Telomerase is thought to increase the radiation resistance of cancer cells by either protecting telomeres from fusion or by its anti-apoptotic functions or by promoting DNA repair through its actions on the chromatin structure (11,34–36,84–87). A telomerase antagonist, imetelstat in combination with radiation and temozolomide had a dramatic effect on cell survival of primary human glioblastoma tumor-initiating cells (45). Telomere targeting with a G-quadruplex ligand, has been recently reported to enhance radiation-induced killing of human glioblastoma cells (44).

The personalization of glioblastoma medicine around telomere profiling in radiation therapy is already under study (88), and could be extended to telomerase activity. Our results showing that telomerase upregulation was not abolished by the PI3K/AKT pathway inhibition, suggests that personalized combined therapies associating PI3K and telomerase inhibitors or telomere G-quadruplex ligands should be considered to improve the radiosensitization in telomerase expressing high-grade gliomas.
